# Usefulness of fluorodeoxyglucose positron emission tomography/computed tomography for detection of a neuroblastic nodule in a ganglioneuroblastoma: a case report

**DOI:** 10.1186/s13256-018-1640-0

**Published:** 2018-05-03

**Authors:** Yuka Takeda, Hideki Sano, Asuka Kawano, Kazuhiro Mochizuki, Nobuhisa Takahashi, Shogo Kobayashi, Yoshihiro Ohara, Kazuhiro Tasaki, Mitusuaki Hosoya, Atsushi Kikuta

**Affiliations:** 1 Department of Pediatric Oncology, Fukushima Medical University Hospital, 1 Hikarigaoka, Fukushima City, Fukushima 960-1295 Japan; 2 Department of Pediatrics, Fukushima Medical University, 1 Hikarigaoka, Fukushima City, Fukushima 960-1295 Japan; 3 Department of Pathology and Laboratory Medicine, Children’s Hospital Los Angeles, 4650 Sunset Boulevard, Los Angeles, CA 90027 USA; 4 Department of Diagnostic Pathology, Fukushima Medical University, 1 Hikarigaoka, Fukushima City, Fukushima, 960-1295 Japan

**Keywords:** FDG PET/CT, Ganglioneuroblastoma, nodular, Ganglioneuroma, Neuroblastoma

## Abstract

**Background:**

Ganglioneuroblastoma, nodular is defined as a composite tumor of biologically distinct clones. The peripheral neuroblastic tumors in this category are characterized by the presence of grossly visible neuroblastoma nodules coexisting with ganglioneuroblastoma, intermixed, or with ganglioneuroma. Making a correct diagnosis of ganglioneuroblastoma, nodular is often difficult by biopsy or partial tumor resection, because the neuroblastic nodule could be hidden and not sampled for pathological examination.

**Case presentation:**

We report a case of a Japanese boy aged 3 years, 8 months, with an unresectable abdominal tumor and elevated vanillylmandelic acid and homovanillic acid levels. The initial biopsy was ganglioneuroma. However, after the second biopsy from a hidden neuroblastoma nodule that was clearly highlighted by fluorodeoxyglucose positron emission tomography/computed tomography, we reached the diagnosis of ganglioneuroblastoma, nodular. Because the nodule demonstrated neuroblastoma, differentiating subtype, with a low mitosis-karyorrhexis index (favorable histology) and nonamplified *MYCN*, the boy was treated according to the intermediate-risk protocol and is now alive and well 4 years after the diagnosis.

**Conclusions:**

This case illustrates the critical role of fluorodeoxyglucose positron emission tomography/computed tomography for detecting a neuroblastoma nodule in a ganglioneuroblastoma.

## Background

Peripheral neuroblastic tumor (pNT), derived from sympathoadrenal lineage of the neural crest, is one of the most common extracranial solid tumors in children. The International Neuroblastoma Pathology Classification (INPC) distinguishes four categories of pNTs as follows: neuroblastoma (NB; Schwannian stroma-poor); ganglioneuroblastoma, intermixed (GNBi; Schwannian stroma-rich); ganglioneuroma (GN; Schwannian stroma-dominant); and ganglioneuroblastoma, nodular (GNBn; composite, Schwannian stroma-dominant/stroma-rich, and stroma-poor) [1, 2]. INPC classifies pNTs into either the favorable or unfavorable histology group, and this classification plays a critical role in patient risk stratification for the Children’s Oncology Group (COG) neuroblastoma clinical trials.

Among these tumor categories, GNBn is defined as a composite tumor of biologically distinct clones. pNTs in this category are characterized by the presence of a grossly visible NB nodule coexisting with GNBi or GN. Making a correct diagnosis of GNBn is often difficult by biopsy or partial tumor resection, because the NB nodule could be hidden and not sampled for pathological examination. Although the nodule is frequently hemorrhagic and/or necrotic, it may not be easily detectable by conventional imaging analyses and ^123^I-metaiodobenzylguanidine (^123^I-MIBG) scintigraphy. However, detecting/sampling the NB nodule for molecular testing and histopathological classification is essential for appropriate clinical management of patients with GNBn.

We report a case of a pediatric patient with a huge and unresectable stage 3 pNT. After the initial biopsy diagnosis of GN, the final diagnosis of GNBn was made on the basis of the result of the second biopsy from the NB nodule, which was clearly identified/located by fluorodeoxyglucose (FDG) positron emission tomography (PET)/computed tomography (CT). Successful clinical management of the patient was accomplished on the basis of the molecular and histopathological characteristics of the NB nodule of this case.

## Case presentation

**Fig. 1:**
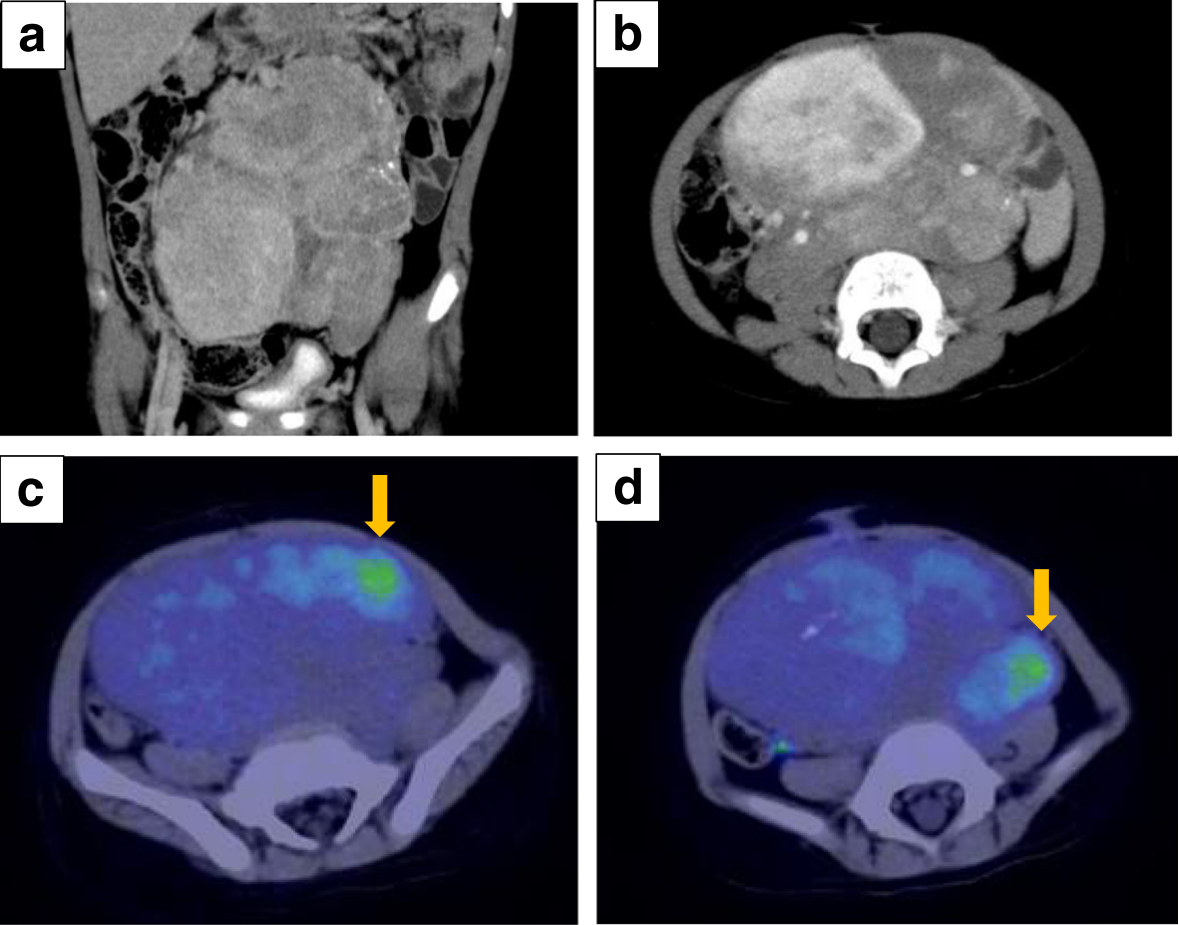
**a** A coronal computed tomographic image showing a huge abdominal tumor extending from the retroperitoneum to the pelvis. **b** Axial contrast-enhanced computed tomography of the abdomen showing heterogeneous enhancement. The tumor shows bilateral encasement by the common iliac artery. **c** and **d** Axial fluorodeoxyglucose positron emission tomography/computed tomography showing abnormal accumulation with a maximum standardized uptake value of 4.20 (*arrows*)

A Japanese boy aged 3 years, 8 months was presented to our institution with marked abdominal distention. CT revealed a huge mass with calcification, measuring 13 cm in diameter, in the median abdominal region. The tumor, encasing bilateral common iliac arteries, was stage 3 according to the International Neuroblastoma Staging System [3] (Fig. 1a, b), and it was unresectable. Whole-body ^123^I-MIBG scintigraphy revealed no metastatic spread. The patient’s urinary catecholamine metabolites were markedly elevated; his vanillylmandelic acid (VMA) level was 48.7 μg/mg creatinine (normal range 4.3–12.1 μg/mg); and his homovanillic acid (HVA) level was 221 μg/mg creatinine (normal range 5.8–18.7 μg/mg).

**Fig. 2:**
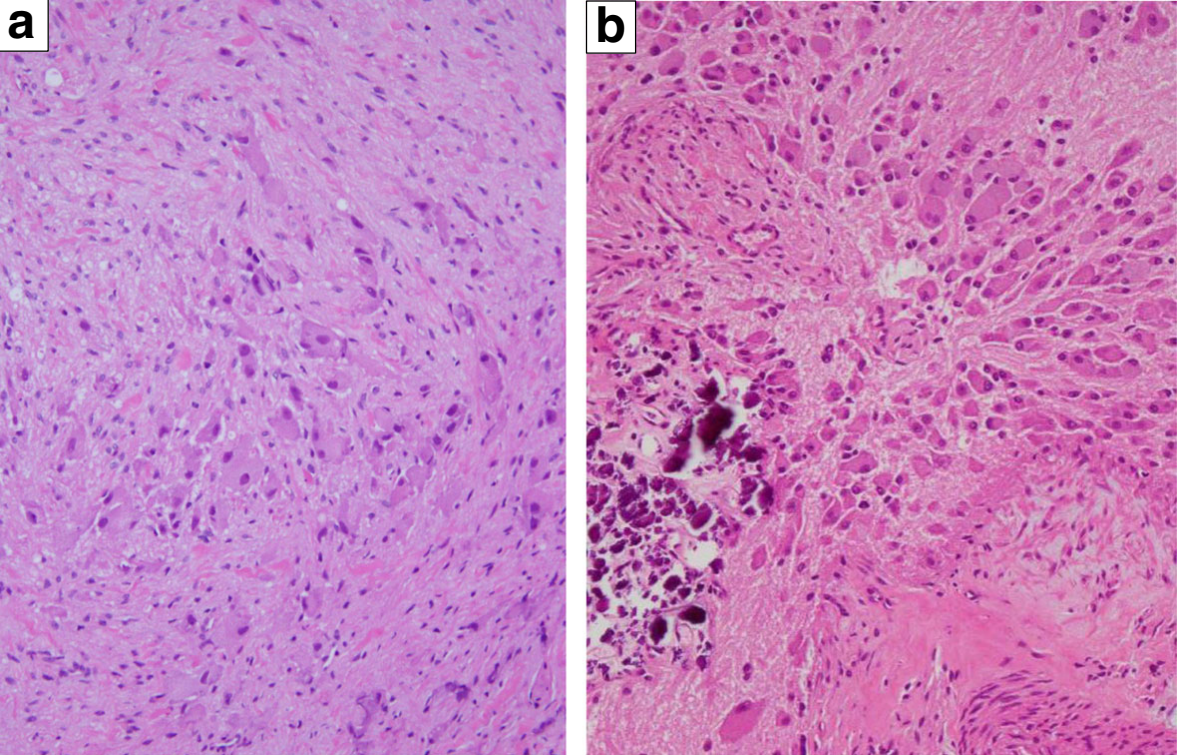
**a** Histopathological study of the initial biopsy revealing ganglioneuroma (Schwannian stroma-dominant), maturing subtype, and favorable histology (FH). Hematoxylin-Eosin (H&E) stain, original magnification ×100. **b** Histopathological study of the second biopsy revealing neuroblastoma (Schwannian stroma-poor), differentiating subtype, low mitosis-karyorrhexis index, and FH. H&E stain, original magnification ×100

**Fig. 3:**
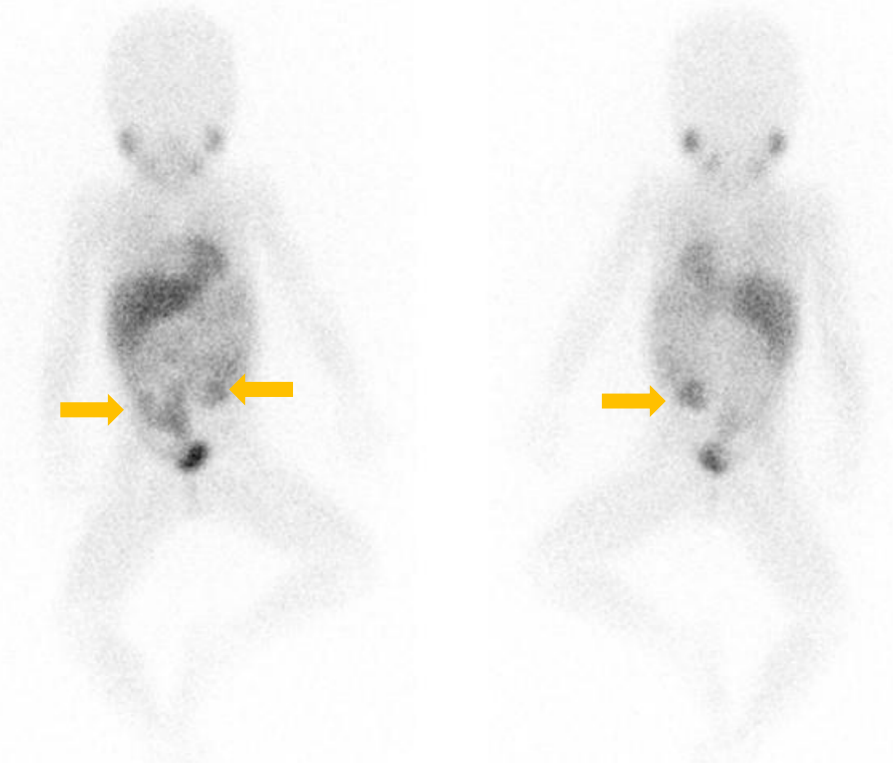
Whole-body ^123^iodine-metaiodobenzylguanidine (^123^I-MIBG) scintigraphy at diagnosis showing partial uptake of ^123^I-MIBG (*arrows*)

The pathological diagnosis based on the initial biopsy was GN (Fig. 2a). Although the highly elevated urinary VMA/HVA levels prompted us to search for a hidden NB clone, imaging analyses, including contrast-enhanced CT, failed to show any nodular formation. The tumor demonstrated a partial uptake of ^123^I-MIBG, but the neuroblastic nodule was difficult to locate (Fig. 3). Further examination using FDG PET/CT, however, revealed the nodular lesion of the NB growth with a maximum standardized uptake value (SUV_max_) of 4.20 in the primary tumor mass (Fig. 1c, d).

The patient subsequently underwent a second biopsy of the nodular lesion, with increased FDG uptake. The biopsy tissue showed an appearance characteristic of NB, differentiating subtype, with a low mitosis-karyorrhexis index (MKI) (< 100/5000 cells) and favorable histology according to the INPC (Fig. 2b). Fluorescence *in situ* hybridization analysis indicated that the *MYNC* oncogene was not amplified in the NB nodule.

According to the COG criteria, the patient was in the intermediate-risk group, and he was treated with one cycle of cyclophosphamide/vincristine/pirarubicin, two cycles of cyclophosphamide/vincristine/carboplatin, and six cycles of cyclophosphamide/vincristine/pirarubicin/cisplatin, in accordance with the protocol of the Japan Neuroblastoma Study Group. At the time of chemotherapy completion, the tumor had not shrunk, and the patient’s urinary VMA/HVA levels remained elevated. Because the GNBn in this case was biologically favorable, we opted for clinical observation without further therapy. Four years after the diagnosis, the patient was doing well with no detectable ^123^I-MIBG avid foci and normalized VMA/HVA levels, whereas the tumor size had slightly increased.

## Discussion

In 1947, Stout *et al*. first described a composite tumor that contained a malignant NB nodule coexisting with ganglioneuromatous tissue [4]. In 1984, Shimada *et al*. clearly defined the morphological characteristics of this category as pNT and named it “stroma-rich (ganglioneuroblastoma), nodular” [5]. In 1999, the International Neuroblastoma Pathology Committee changed the name of this category to “Ganglioneuroblastoma, nodular (GNBn)” [1, 2]. In 2000, Umehara *et al*. identified two prognostic groups in this category, and the results were confirmed by the committee in 2003 [6]. Briefly, the “favorable versus unfavorable” histology classification according to the INPC, based on the age-linked morphological evaluation (grade of neuroblastic differentiation and MKI), that was applied to the NB nodule significantly distinguishes better and worse prognoses, respectively, of patients with GNBn. In 2012, Angelini *et al.* reported the results of the study of the International Neuroblastoma Risk Group, and the event-free and overall survival rates of GNBn tumors in the favorable histology group were 87 ± 8% and 91 ± 6%, respectively, whereas those in the unfavorable histology group were 45 ± 6% and 61 ± 5%, respectively [7].

For clinical management of patients with pNT, detection/sampling of the NB nodule is critical when present and coexisting with GNBi or GN. However, in our routine practice, we could miss the nodule by biopsy or partial resection of the tumors that show only GN or GNBi histology. Currently, the COG Neuroblastoma Pathology Reference Laboratory recommends a disclaimer of “based on review of limited material” in the diagnosis line after “GN or GNBi, favorable histology” in the surgical pathology report. In this clinical situation, the COG Neuroblastoma Pathology Reference Laboratory also recommends measuring VMA/HVA levels, because hidden NB nodules can often be the source of high elevation of these markers. By contrast, GN and GNBi tumors, predominantly or mainly composed of Schwannian stroma, usually do not produce higher levels of VMA/HVA.

In our patient’s case, we conducted imaging analyses for detection of the NB nodule. Conventional CT and magnetic resonance imaging were not useful for this purpose. Although ^123^I-MIBG scintigraphy has been widely used for the diagnosis of pNTs, it was also not helpful for identifying the NB nodule in the otherwise GN background of our patient. As reported by Decarolis *et al*., 24.5% of GN tumors and 61.1% of GNBi tumors showed MIBG uptake, so that any NB nodule, when present, may not be highlighted by this scintigraphy method [8]. However, we detected/highlighted the NB nodule in our patient by using FDG PET/CT, on the basis of the difference in FDG accumulation as follows: midlevel FDG accumulation (SUV_max_ 4.10) in the NB nodule [9] in contrast to the lower FDG accumulation (SUV_max_ 2.02) in GN tissue [10].

Management of residual tumors after completion of chemotherapy has been controversial. Marachelian *et al*. [11] and Iehara *et al.* [12] demonstrated that residual tumors in patients with intermediate-risk pNTs were not associated with tumor progression, regardless of ^123^I-MIBG avidity and/or elevated catecholamines. Considering the characteristics of the NB nodule (favorable histology and nonamplified *MYCN*) in our patient, we adopted an observational approach to wait for age-linked tumor maturation, regardless of persistently high levels of catecholamines. As clarified by Decrarolis *et al.*, the large GN portion of this tumor, which is composed of abundant Schwannian stromal cells, would not shrink after chemotherapy [8]. As suggested by Nishihira *et al*., incremental increase in tumor volume may result from the proliferation of Schwannian stroma cells in the course of the maturational step of a biologically favorable NB nodule to GN [13]. After 4 years of follow-up, our patient was still alive and well with normalized catecholamine levels.

## Conclusions

In summary, we report a case of a pediatric patient with unresectable stage 3 GNBn. Biopsy of the NB nodule, which was clearly highlighted by FDG PET/CT, led us to successful clinical management of the patient. Our report suggests the potential usefulness of FDG PET/CT rather than ^123^I-MIBG scintigraphy for detection of NB nodules in GNBn.

## Abbreviations

COG: Children’s Oncology Group
CT: Computed tomography
FDG: Fluorodeoxyglucose
FH: Favorable histology
GN: Ganglioneuroma
GNBi: Ganglioneuroblastoma, intermixed
GNBn: Ganglioneuroblastoma, nodular
HVA: Homovanillic acid
^123^I-MIBG: ^123^Iodine-metaiodobenzylguanidine
INPC: International Neuroblastoma Pathology Classification
MKI: Mitosis-karyorrhexis index
NB: Neuroblastoma
PET: Positron emission tomography
pNT: Peripheral neuroblastic tumor
SUV_max_: Maximum standardized uptake value
VMA: Vanillylmandelic acid

## References

[1] Shimada H, Ambros IM, Dehner LP, Hata J, Joshi VV, Roald B, *et al*. The International Neuroblastoma Pathology Classification (the Shimada System). Cancer. 1999;86:364–72. 10421273

[2] Shimada H, Ambros IM, Dehner LP, Hata J, Joshi VV, Roald B. Terminology and morphologic criteria of neuroblastic tumors: recommendations by the International Neuroblastoma Pathology Committee. Cancer. 1999;86:349–63. 10421272

[3] Monclair T, Brodeur GM, Ambros PF, Brisse HJ, Cecchetto G, Holmes K, *et al*. The International Neuroblastoma Risk Group (INRG) staging system: an INRG Task Force report. J Clin Oncol. 2009;27:298–303. 10.1200/JCO.2008.16.6876 PMC2650389 19047290

[4] Stout AP. Ganglioneuroma of the sympathetic nervous system. Surg Gynecol Obstet. 1947;84:101–10. 20278169

[5] Shimada H, Chatten J, Newton WA Jr, Sachs N, Hamoudi AB, Chiba T, *et al*. Histopathologic prognostic factors in neuroblastic tumors: definition of subtypes of ganglioneuroblastoma and an age-linked classification of neuroblastomas. J Natl Cancer Inst. 1984;73:405–16. 10.1093/jnci/73.2.405 6589432

[6] Umehara S, Nakagawa A, Matthay KK, Lukens JN, Seeger RC, Stram DO, *et al*. Histopathology defines prognostic subsets of ganglioneuroblastoma, nodular. Cancer. 2000;89:1150–61. 10964346

[7] Angelini P, London WB, Cohn SL, Pearson AD, Matthay KK, Monclair T, *et al*. Characteristics and outcome of patients with ganglioneuroblastoma, nodular subtype: a report from the INRG project. Eur J Cancer. 2012;48:1185–91. 10.1016/j.ejca.2011.10.037 22137163

[8] Decarolis B, Simon T, Krug B, Leuschner I, Vokuhl C, Kaatsch P, von Schweinitz D, Klingebiel T, Mueller I, Schweigerer L, Berthold F, Hero B. Treatment and outcome of ganglioneuroma and ganglioneuroblastoma intermixed. BMC Cancer. 2016;16:542. 10.1186/s12885-016-2513-9 PMC4964292 27465021

[9] Liu YL, Lu MY, Chang HH, Lu CC, Lin DT, Jou ST, *et al*. Diagnostic FDG and FDOPA positron emission tomography scans distinguish the genomic type and treatment outcome of neuroblastoma. Oncotarget. 2016;7(14):18774–86. 10.18632/oncotarget.7933 PMC4951328 26959748

[10] Miyake M, Tateishi U, Maeda T, Arai Y, Seki K, Hasegawa T, et al. A case of ganglioneuroma presenting abnormal FDG uptake. Ann Nucl Med. 2006;20(5):357–60. 10.1007/BF02987247 16878708

[11] Marachelian A, Shimada H, Sano H, Jackson H, Stein J, Sposto R, *et al*. The significance of serial histopathology in a residual mass for outcome of intermediate risk stage 3 neuroblastoma. Pediatr Blood Cancer. 2012;58:675–81. 10.1002/pbc.23250 22493777

[12] Iehara T, Yagyu S, Tsuchiya K, Kuwahara Y, Miyachi M, Tajiri T, *et al*. Residual tumor in cases of intermediate-risk neuroblastoma did not influence the prognosis. Jpn J Clin Oncol. 2016;46:661–6. 10.1093/jjco/hyw050 27207883

[13] Nishihira H, Toyoda Y, Tanaka Y, Ijiri R, Aida N, Takeuchi M, *et al*. Natural course of neuroblastoma detected by mass screening: a 5-year prospective study at a single institution. J Clin Oncol. 2000;18:3012–7. 10.1200/JCO.2000.18.16.3012 10944135

